# Prospective Surveillance with Compression for Subclinical Lymphedema: Symptoms, Skin, and Quality-of-Life Outcomes

**DOI:** 10.1089/lrb.2022.0020

**Published:** 2023-06-23

**Authors:** Mary S. Dietrich, Katrina Gaitatzis, Louise Koelmeyer, John Boyages, Vandana G. Abramson, Sarah A. McLaughlin, Nicholas Ngui, Elisabeth Elder, James French, Jeremy Hsu, T. Michael Hughes, Deonni P. Stolldorf, Chirag Shah, Sheila H. Ridner

**Affiliations:** ^1^ Vanderbilt University School of Nursing, Nashville, Tennessee, USA.; ^2^ Department of Biostatistics, Vanderbilt University School of Medicine, Nashville, Tennessee, USA.; ^3^ Australian Lymphoedema Education, Research, and Treatment (ALERT) Program, Department of Health Sciences, Macquarie University Macquarie Park, Sydney, Australia.; ^4^ Sydney Adventist Hospital Integrated Cancer Centre, ICON Cancer Centre, Wahroonga, Australia.; ^5^ Division of Hematology/Oncology, Vanderbilt-Ingram Cancer Center, Nashville, Tennessee, USA.; ^6^ Mayo Clinic, Jacksonville, Florida, USA.; ^7^ Northern Surgical Oncology, Sydney Adventist Hospital, Wahroonga, Australia.; ^8^ Westmead Breast Cancer Center Institute, Westmead Hospital, Westmead, Australia.; ^9^ Department of Surgery, The University of Sydney, Sydney, Australia.; ^10^ Lakeside Specialist Breast Clinic, Lakeview Private Hospital Norwest, Norwest, Australia.; ^11^ Department of Clinical Medicine, Faculty of Medicine, Health and Human Sciences, Macquarie University, Macquarie Park, Australia.; ^12^ Sydney Adventist Hospital Clinical School, College of Health and Medicine, Australian National University, Canberra, Australia.; ^13^ Taussig Cancer Institute, Cleveland Clinic, Cleveland, Ohio, USA.

**Keywords:** breast cancer, subclinical lymphedema, symptoms, skin, quality of life

## Abstract

**Background:**

Patients underwent a compression (sleeve and gauntlet) intervention for subclinical breast cancer-related lymphedema (S-BCRL). Physical, emotional, and quality-of-life (QoL) outcomes were examined. Associations of change in extracellular fluid alone through bioimpedance spectroscopy (BIS) or change in whole-arm volume through tape measure with the outcomes at time of S-BCRL were explored.

**Methods and Results:**

We enrolled newly diagnosed nonmetastatic breast cancer patients for surveillance up to 36 months postoperatively. Upon detection of S-BCRL, a 28-day compression intervention was initiated. Data were obtained through physical examination/measurement and self-report instruments: skin examination, Lymphedema Symptom Intensity and Distress Survey-Arm, and Functional Assessment of Cancer Therapy General (FACT-G), Breast (FACT-B), and FACT-B+4.

Improvements with intervention were observed in the proportion of patients reporting symptom scores ≥3 in function (Cohen's *d* = −0.46, *p* < 0.01), in biobehavioral (Cohen's *d* = −0.30, *p* < 0.05), maximum number of skin conditions (Cohen's *d* = −0.34, *p* < 0.05. 3), FACT-B (Cohen's *d* = 0.52, *p* < 0.01), and FACT-B + four (Cohen's *d* = −0.42, *p* < 0.01). At the study endpoint, compared with those who did not progress, chronic breast cancer-related lymphedema (C-BCRL) progressing patients had higher overall symptom scores (*p* = 0.037), more skin conditions (*p* = 0.009), and lower total FACT-G and FACT-B scores (*p* < 0.05). At the time of S-BCRL, detection of greater BIS unit change correlated with higher symptom, skin condition, and QoL values. Greater whole-arm volume change correlated with higher FACT-B+4 scores (all *p* < 0.05).

**Conclusions:**

Prospective surveillance, symptom assessment, and compression intervention promote low progression rates from S-BCRL to C-BCRL and as such reduce symptom burden. This closed study is registered with ClinicalTrials.gov NCT02167659

## Introduction

Growing emphasis has been placed on the prevention, diagnosis, and management of chronic breast cancer-related lymphedema (C-BCRL).^1^ Traditionally, C-BCRL has been diagnosed with circumference tape measurements (TM) and/or self-reported swelling.^1^ When using TM, a ≥ 10% volume difference between at-risk and non-at-risk arms, or change from presurgical baseline of ≥10% arm volume change from baseline has served as the C-BCRL diagnostic threshold.^2^ Established, C-BCRL creates a complex series of physical and emotional morbidities,^3,4^ often leading to a compromised quality of life (QoL).^3–5^ In addition, scientific evidence demonstrates that C-BCRL-associated inflammation directly compromises the skin, leading to episodes of cellulitis that require antibiotic therapy.^5^ Taken together, the impact of C-BCRL on QoL is significant.^6^

An emerging field of research addresses subclinical BCRL (S-BCRL), a condition that occurs when lymph transport is compromised, but visible swelling is absent.^7–9^ Bioimpedance spectroscopy (BIS) has a high sensitivity for detection of S-BCRL when there is a ≥ 6.5 L-Dex unit change from the pretreatment baseline.^9–12^ Correspondingly, a ≥ 5% to <10% arm volume change from baseline, when calculated using a tape measure, may represent S-BCRL.^2^ Detection of early-onset S-BCRL through prospective surveillance, when paired with a brief compression intervention, lowers rates of C-BCRL.^8,9^ Thus, patients undergoing prospective surveillance may potentially avoid C-BCRL and the associated morbidities.

Our previous work^9^ examined which physiological process used as an S-BCRL intervention trigger best positioned a patient to reduce the development of C-BCRL when undergoing an intervention for S-BCRL. Triggers included extracellular fluid change measured by a well-established, reliable, and valid BIS methodology or whole-arm volume change measured by well-established reliable and valid methods of TM methodology. Evidence regarding associated morbidities such as physical, emotional, and QoL issues within the context of prospective surveillance with S-BCRL intervention is scant. Therefore, a secondary aim was to examine the clinical outcomes other than swelling, in patients who received a compression intervention for S-BCRL.

Initially we conducted a secondary analysis of physical, emotional, and QoL data in a subset of 508 women enrolled in the parent randomized clinical trial who were 24 months postbreast cancer surgery.^13^ In that analysis, patients were censored immediately before intervention when S-BCRL was identified. We now extend that work. Specifically, the purpose of this secondary analysis was to examine the physical, emotional, and QoL outcomes within the context of prospective surveillance for S-BCRL with a subsequent intervention. Associations with underlying physiological processes used as detectors for S-BCRL on those outcomes were explored.

## Materials and Methods

### Design, settings, and patients

Data were obtained from a multisite, international, stratified, randomized clinical trial.^9^ Data from assessments conducted at the time of S-BCRL detection, immediately following intervention, and study endpoint were examined in this analysis. Site-specific regulatory and ethics approval was attained before parent study onset, and it was registered at ClinicalTrials.gov. Patients were screened, recruited, and enrolled before treatment of newly diagnosed breast cancer from June 24, 2014, through September 11, 2018.

Those patients who met the S-BCRL thresholds (via TM or BIS) for the intervention before progression to C-BCRL comprised the sample for this study (*N* = 209). Of those, 189 had completed symptom, skin condition, and QoL assessments before undergoing and completing the intervention and at study endpoint. They are included in this analysis.

### S-BCRL treatment

The interventional treatment consisting of a 23–32 mmHg, off-the-shelf medi Harmony^®^ circular knit or medi flat knit custom compression sleeve and gauntlet was initiated when an S-BCRL trigger occurred. This has been previously described.^13,14^ If the immediate postintervention assessment, or any subsequent assessment, identified a ≥ 10% arm volume difference on TM, a referral to complex decongestive physiotherapy for C-BCRL progression was initiated. Referred patients were removed from the study.

### Data collection

The Research Electronic Data Capture (REDCap) database environment was used for data collection and management.^15^ The following data collection tools and methods were used:

#### Demographics, cancer, and medical history

Self-reported demographic data, extracted medical record cancer stage, and treatment and medical history data were obtained.

#### L-Dex U400

BIS detected changes in extracellular fluid.^16^ Measurements were made following the manufacturer's recommended protocol. An L-Dex^®^ value was generated by the device and recorded.

#### Gulick II tape measure

A weighted tape was used to measure arm circumference. The protocol required arms to be aligned with a premarked arm board to ensure consistent measurement locations across time points.^17^ Measurements were entered into a database. Volume was calculated using a truncated cone formula.

#### Skin assessment checklist

Trained research staff examined both arms and documented findings on a standardized 17-item checklist (e.g., hard, soft, warm, cold, cracked).^3,18^

#### Lymphedema Symptom Intensity and Distress Survey-Arm

This self-report, 30-item survey, consisting of 7 symptom clusters (soft tissue, neurological sensation, function, biobehavioral, resource, sexuality, and activity), was used to document symptoms.^3,4^ Patients indicated the presence of a symptom (“yes” or “no”), and if “yes,” separately rated intensity and associated distress on two separate 1–5-point scales. In this study, average Cronbach's alpha values were overall 0.88, soft tissue 0.71, neurological sensation 0.81, function 0.65, biobehavioral 0.82, resource 0.88, sexuality 0.76, and activity 0.82.

#### Functional Assessment of Cancer Therapy Breast Plus 4

The 36-item self-report Functional Assessment of Cancer Therapy Breast (FACT-B) assessed QoL across five domains. These included physical well-being, social/family well-being, emotional well-being, functional well-being, and additional concerns using a 0- to 4-point scale.^19^ The previously validated FACT-B Plus 4-item scale addressed arm-specific QoL concerns (e.g., painful arm movement, poor range of motion, numbness, stiffness).^20^ In this study**,** average Cronbach's alpha values were as follows: physical 0.80, social 0.88, emotional 0.66, functional 0.88, G-total 0.91, additional concerns 0.66, B-total 0.92, and B + 4 0.79.

### Data analysis

Data analyses were conducted using IBM SPSS Statistics 28. Descriptive statistical summaries were generated for demographic and treatment characteristics. Comparisons between the groups who did and did not progress to C-BCRL were conducted using Mann–Whitney and Chi-Square or Exact tests. Summaries of the symptom, skin, and QoL scores were generated at the time of detection of S-BCRL, post-treatment for S-BCRL, and at study endpoint (last assessment in the study). Correlations of L-Dex and arm volume changes with each measure at time of detection were examined using Spearman's rho coefficients. Wilcoxon Signed Rank tests with the respective effect statistic (Cohen's *d*) were used to assess the effect of treatment on symptom, skin, and QoL scores. A maximum alpha of 0.05 was used for determinations of statistical significance (*p* < 0.05).

## Results

### Participant characteristics

The overall sample (*N* = 189) of patients with S-BCRL detected through change in extracellular fluid only (*n* = 81) or whole-arm volume (*n* = 108) (Table 1) was entirely female, median age of 58.3 years, predominantly white (78%), and with a median 16.0 years of education. Approximately half (51%) dwelled in suburban areas, with the remaining split between urban and rural. Within that sample, 30 patients (16%) progressed to C-BCRL within the study time period. Those patients had statistically significantly fewer years of education than did those who did not progress to C-BCRL (median 13.0 vs. 16.0, *p* = 0.027), comprised a higher percentage who self-defined as black (16.7% vs. 5.7%), and had a higher percentage of Stage III cancer (36.7% vs. 8.2%) (all *p* < 0.05; Table 1). S-BCRL was detected a median 7.1 months postinitial breast cancer surgery (interquartile range [IQR]: 4.0, 17.6, min = 2, max = 35).

**Table 1.** Sociodemographic and Environmental Characteristics of Sample for Subclinical Breast Cancer-Related Lymphedema Detection (*N* = 189)

Characteristic	All detected for S-BCRL (*N* = 189)	Never progressed (*N* = 159)	Progressed to C-BCRL (*N* = 30)	*p*-value^*^
Age, median (IQR)	58.3 (51, 67)	58.2 (51, 61)	60.7 (49, 70)	0.387
Years of education, median (IQR)	16.0 (12, 16)	16.0 (12, 17)	13.0 (12, 16)	0.027
Missing	1	1	0	
Race, *n* (%)				0.025
Asian	14 (7.5)	14 (8.9)	0 (0.0)	
Black or African American	14 (7.5)	9 (5.7)^a^	5 (16.7)^b^	
White	146 (78.0)	121 (77.1)	25 (83.3)	
Multiracial or other	13 (7.0)	13 (8.3)	0 (0.0)	
Missing	2	2	0	
Marital status, *n* (%)				0.118
Single	30 (16.2)	24 (15.5)	6 (20.0)	
Married/partnered	140 (75.7)	121 (78.0)	19 (63.3)	
Widowed/separated	15 (8.1)	10 (6.5)	5 (16.7)	
Missing	4	4	0	
Area of residence, *n* (%)				0.437
City/urban	49 (26.1)	44 (27.8)	5 (16.7)	
Suburban	96 (51.0)	79 (50.0)	17 (56.6)	
Rural	43 (22.9)	35 (22.2)	8 (26.7)	
Missing	1	1	0	
Stage of cancer, *n* (%)				
0 (DCIS)	6 (3.2)	6 (3.8)	0 (0.0)	<0.001
I	95 (50.2)	85 (53.4)^a^	10 (33.3)^b^	
II	64 (33.9)	55 (34.6)	9 (30.0)	
III	24 (12.7)	13 (8.2)^a^	11 (36.7)^b^	

All participants indicated female gender.

^*^ Mann–Whitney test (age, education) or Chi-Square Test of Independence (all other variables).

a,b Specific cells that are statistically significantly different, Bonferroni-corrected, *p* < 0.05.

C-BCRL, chronic breast cancer-related lymphedema; DCIS, ductal carcinoma *in situ*; IQR, interquartile range; S-BCRL, subclinical breast cancer-related lymphedema.

The majority of all of the patients had breast conserving surgery (70.9%) and SLNB only (67.7%). Compared with those who did not progress to C-BCRL, a higher percentage of those who did progress to C-BCRL had ALND (56.7% vs. 25.8%) and some type of chemotherapy (73.3% vs. 47.1%) (both *p* < 0.05; Table 2). For comparison purposes with the entire sample enrolled in the randomized study, detailed demographic and clinical summaries are in previously published work.^8,9,12^

**Table 2.** Breast Cancer Treatment Characteristics of Sample for Subclinical Breast Cancer-Related Lymphedema Detection (*N* = 189)

	All detected for S-BCRL (*N* = 189),* n* (%)	Never progressed (*N* = 159),* n* (%)	Progressed to C-BCRL (*N* = 30),* n* (%)	*p-*value^*^
Treatment characteristics				
Type of surgery				0.381
Breast conservation	134 (70.9)	115 (72.3)	19 (63.3)	
Mastectomy	55 (29.1)	44 (27.7)	11 (36.7)	
Axillary surgery				0.003
None	3 (1.6)	3 (1.9)	0 (0.0)	
ALND	58 (30.7)	41 (25.8)^a^	17 (56.7)^b^	
SLNB only	128 (67.7)	115 (72.3)^a^	13 (43.3)^b^	
Chemotherapy				0.044
None	92 (48.7)	84 (52.9)^a^	8 (26.7)^b^	
Neoadjuvant only	13 (6.9)	10 (6.3)	3 (10.0)	
Adjuvant	72 (38.1)	57 (35.8)	15 (50.0)	
Both	12 (6.3)	8 (5.0)	4 (13.3)	
If chemotherapy, type	(*n* = 97)	(*n* = 75)	(*n* = 22)	0.999
Any taxane	91 (93.8)	158 (89.8)	150 (87.2)	
Other (not taxane)	6 (6.2)	18 (10.2)	22 (12.8)	
Radiation therapy				0.274
No	30 (15.9)	23 (14.5)	7 (23.3)	
Yes	159 (84.1)	136 (85.5)	23 (76.7)	
Endocrine therapy				0.174
No	49 (26.1)	38 (24.1)	11 (36.7)	
Yes	139 (73.9)	120 (75.9)	19 (63.3)	
Missing	1	1	0	
Complete treatment				0.218
Surgery only	18 (9.7)	16 (10.3)	2 (7.0)	
Surgery + radiation (not RNI)	63 (34.1)	58 (37.2)	5 (17.2)	
Surgery + RNI	10 (5.4)	9 (5.8)	1 (3.4)	
Surgery + chemo (taxane)	15 (8.1)	10 (6.4)	5 (17.2)	
Surgery + chemo (not taxane)	0 (0.0)	0 (0.0)	0 (0.0)	
Surgery + radiation (not RNI) + chemo (taxane)	26 (14.1)	21 (13.5)	5 (17.2)	
Surgery + radiation (not RNI) + chemo (not taxane)	6 (3.2)	5 (3.2)	1 (3.4)	
Surgery + RNI + chemo (taxane)	47 (25.4)	37 (23.6)	10 (34.6)	
Surgery + RNI + chemo (not taxane)	0 (0.0)	0 (0.0)	0 (0.0)	
Missing	4	3	1	

All participants had surgery as per inclusion criteria.

^*^ Fisher exact test or chi-square test of independence.

a,b Specific cells that are statistically significantly different, Bonferroni-corrected, *p* < 0.05.

ALND, axillary lymph node dissection; RNI, regional node; SLNB, sentinel lymph node biopsy.

### Primary health outcomes

#### Symptoms: Lymphedema Symptom Intensity and Distress Survey-Arm

As summarized in Table 3, in general, symptom burden self-reports were very low in our sample with median values being <1.0 of a possible 10 for all symptom clusters and at both times of assessment. A higher level of change in L-Dex units at the time of detection from baseline was statistically significantly correlated with higher overall symptom scores, as well as with higher specific soft tissue, neurologic, and sexuality symptom cluster scores (*p* < 0.05). No significant correlations with the symptoms scores were observed for the changes in total arm volume values (*p* > 0.10) (Table 3).

**Table 3.** Summaries of Lymphedema Symptom Intensity and Distress Survey-Arm Scores and Number of Skin Conditions in Affected Arm Pre- and Postreferral for Subclinical Lymphedema Treatment (*N* = 189)

LSIDS-A cluster	Correlation with scores at detection		Detection	Post Tx	
	L-Dex unit change, r_s_ (*p*-value)	Arm volume change, r_s_ (*p*-value)	Median (IQR) [max, % ≥ 3]	Median (IQR) [max, % ≥ 3]	Cohen's* d*
			*n* = 183		
Overall	0.21 (0.004)^**^	0.05 (0.509)	0.5 (0.1, 1.3) [6.0, 3.8]	0.5 (0.1, 1.2) [5.3, 2.2]	−0.28
			*n* = 184		
Soft tissue	0.17 (0.022)^*^	0.06 (0.391)	0.0 (0.0, 0.5) [8.0, 8.2]	0.0 (0.0, 1.5) [5.5, 7.1]	−0.09
			*n* = 186		
Neurological	0.17 (0.017)^*^	0.10 (0.195)	0.0 (0.0, 0.6) [6.0, 5.4]	0.0 (0.0, 0.6) [6.3, 4.3]	−0.01
			*n* = 186		
Function	0.04 (0.614)	0.03 (0.727)	0.0 (0.0, 0.0) [8.0, 9.1]	0.0 (0.0, 0.0) [7.0, 3.2]	−0.46^**^
			*n* = 186		
Biobehavioral	0.13 (0.077)	0.02 (0.753)	0.6 (0.0, 1.6) [6.3, 10.2]	0.5 (0.0, 1.4) [7.8, 7.5]	−0.30^*^
			*n* = 185		
Resource	0.07 (.373)	−0.11 (0.126)	0.0 (0.0, 0.0) [10.0, 3.2]	0.0 (0.0, 0.0) [9.0, 3.8]	−0.08
			*n* = 103		
Sexuality	0.18 (0.048)^*^	−0.01 (0.970)	0.0 (0.0, 1.4) [7.0, 16.5]	0.5 (0.0, 0.0) [9.3, 10.7]	−0.15
			*n* = 186		
Activity	0.06 (0.451)	0.03 (0.649)	0.0 (0.0, 2.0) [10.0, 19.4]	0.0 (0.0, 2.0) [10.0, 19.4]	−0.03
			*Median (IQR, max)*	*Median (IQR, max)*	
			*n* = 189		
No. of skin conditions in affected arm (max = 17)	0.19 (0.008)^**^	0.06 (0.429)	0.0 (0.0, 1.0, max = 9)	0.0 (0.0, 1.0, max = 6)	−0.34^*^

^*^ *p* < 0.05; ^**^p < 0.01.

LSIDS-A, Lymphedema Symptom Intensity and Distress Survey-Arm.

At the time of S-BCRL detection, ∼19.4% of the patients reported scores of at least 3 out of 10 for the Activity cluster of symptoms (give up hobbies, decrease social activity, decrease physical activity). Approximately 16.7% of the S-BCRL subsample completing the Sexuality score (*n* = 103) reporting scores of ≥3 for that cluster that includes lack of interest in sex, partner lack of interest, decrease sexual activity). Neither of those distributions of scores was significantly changed by the intervention (*p* > 0.40, Activity: Cohen's *d* = −0.03, Sexuality: Cohen's *d* = −0.15). Where observed, the apparent effects of the S-BCRL treatment were for those with elevated scores in the Function (move arm side-to-side, raise arm above head) and Behavioral (sadness, anger, lack self-confidence, appearance concerns, misunderstood by significant other, less sexually attractive, loss of body confidence, fatigue, sleep loss) clusters of symptoms (*p* < 0.05).

Approximately 9.1% of the patients at the time of S-BCRL detection reported Function scores ≥3, with a maximum score within the sample of 8.0. Post-treatment, those respective values were reduced to only 3.2% having scores ≥3 with a maximum score of 7.0 (Cohen's *d* = −0.46, *p* < 0.01). The effect for the Biobehavioral symptom reports was not as strong, yet 10.2% of the patients had scores ≥3 at S-BCRL detection with that being reduced to 7.5% post-treatment (Cohen's *d* = −0.30, *p* < 0.05) (Table 3).

#### Skin conditions

The number of skin conditions in the affected arm from the assessments at time of S-BCRL detection and immediately postintervention is also summarized in Table 3. A greater increase in L-Dex units from baseline was statistically significantly correlated with a higher number of conditions (*r_s_* = 0.19, *p* = 0.008); such a correlation was not observed with total arm volume changes (*r_s_* = 0.06, *p* = 0.429).

As with the symptom burden self-reports, the number of skin conditions was generally very low in our sample. The most commonly reported conditions at the time of S-BCRL detection were “dry/flaky” (*n* = 22, 11.6%), “red in color” (*n* = 20, 10.6%), “scabs” (*n* = 14, 7.4%), and “raised lumps” (*n* = 14, 7.4%). Regardless of time of assessment, the median number of symptoms was 0.0, with 81% of the patients having ≤1 condition at the time of detection and 89% having ≤1 condition post-treatment. Nevertheless, the intervention for S-BCRL had an apparent effect for those patients with a higher number of conditions. At the time S-BCRL detection, the maximum number of conditions for any patient in the sample was 9, at post-treatment, that respective value was reduced to 6 (Cohen's *d* = −0.34, *p* < 0.05) (Table 3).

#### Quality of life

Finally, as summarized in Table 4, a greater increase in L-Dex units from baseline was statistically significantly correlated with lower FACT physical scores (*r_s_* = −0.22, *p* = 0.002), while a greater increase in total arm volume from before BC treatment was correlated with higher FACT-B+4 scores (*r_s_* = 0.20, *p* = 0.006). Consistent with the symptom reports and skin conditions, QoL reports were generally in the upper portion of the possible range of scores so that apparent effects of the intervention for S-BCRL were for those in the lower portion of the sample distribution at the time of detection. Those effects were significant for the Physical and the Breast +4 subscale. There was a statistically significant increase in the Physical scores between the time of detection and immediately post-treatment (Cohen's *d* = 0.52, *p* < 0.01) and a decrease in the Breast +4 reports (Cohen's *d* = −0.42, *p* < 0.01) (Table 4).

**Table 4.** Summaries of Functional Assessment of Cancer Therapy Scores Pre- and Postreferral for Subclinical Lymphedema Treatment (*N* = 189)

	Correlation with scores at S-BCRL detection		Detection	Post Tx	
FACT score	L-Dex unit change, r_s_ (*p*-value)	Arm volume change,* r_s_* (*p*-value)	Median (IQR)	Median (IQR)]	Cohen's* d*
			*n* = 186		
Physical (possible range: 0–28)^a^	−0.22 (0.002)^**^	−0.04 (0.598)	25.0 (21.0, 27.0)	25.0 (22.7, 27.0)	0.52^**^
			*n* = 186		
Social (possible range: 0–28)^a^	−0.01 (0.865)	0.03 (.639)	24.8 (21.0, 28.0)	25.0 (22.7, 27.0)	<0.01
			*n* = 186		
Emotional (possible range: 0–24)^a^	−0.09 (0.215)	−0.10 (0.165)	21.0 (19.0, 23.0)	20.0 (19.0, 23.0)	0.11
			*n* = 186		
Functional (possible range: 0–28)^a^	−0.14 (0.051)	−0.05 (0.534)	21.0 (18.0, 26.0)	22.0 (17.0, 26.0)	0.12
			*n* = 186		
B-Subscale (possible range: 0–36)^a^	−0.08 (0.261)	−0.10 (0.163)	27.0 (22.7, 31.0)	27.5 (24.0, 30.0)	0.25
			*n* = 186		
FACT-G total (possible range: 0–108)^a^	−0.14 (0.058)	−0.06 (0.388)	90.4 (79.7, 100.0)	91.2 (78.9, 99.8)	0.33^*^
			*n* = 186		
FACT-B total (possible range: 0–144)^a^	−0.14 (0.052)	−0.07 (0.325)	117.2 (102.6, 128.3)	117.7 (104.0, 129.2)	0.29^*^
			*n* = 186		
B + 4 (possible range: 0–16)^b^	0.08 (0.271)	0.20 (0.006)^**^	0.0 (0.0, 3.0)	0.0 (0.0, 2.0)	−0.42^**^

^a^ Higher score indicates better QOL.

^b^ Higher score indicates more B + 4 symptoms.

^*^ *p* < 0.05; ^**^p < 0.01.

FACT-B, Functional Assessment of Cancer Therapy Breast; FACT-G, Functional Assessment of Cancer Therapy General.

### Primary health outcomes at study endpoint post-lymphedema treatment

Follow-up time in our sample of patients with S-BCRL detected and subsequent treatment was a median 17.0 months after completion of that treatment (IQR: 4.9, 28.0, min = 0, max = 34). Of the 189 patients with S-BCRL, 187 had a skin assessment completed; of those, 185 completed the FACT and most of the Lymphedema Symptom Intensity and Distress Survey-Arm (LSIDS-A) measures. Summaries and comparisons of the self-reported symptoms and QoL, as well as the number of skin conditions at study endpoint for the group of patients progressing to C-BCRL (*n* = 30) and those who did not (*n* = 157), are presented in Tables 5 and 6.

**Table 5.** Summaries of Lymphedema Symptom Intensity and Distress Survey-Arm Scores and Number of Skin Conditions in Affected Arm at Study Endpoint by Progression Group (*N* = 187)

LSIDS-A cluster	Scores				Scores ≥3	
	S-BCRL only	Progression to C-BCRL		S-BCRL only	Progression to C-BCRL	
	Median, max (IQR)	Median, max (IQR)	*p*-value*^a^*	*n *(%)	*n *(%)	*p*-value*^b^*
	*n* = 152	*n* = 30		*n* = 152	*n* = 30	
Overall	0.3, 3.2 (0.0, 0.9)	0.5, 5.3 (0.2, 1.4)	0.037	2 (1.3)	4 (13.3)	0.007
	*n* = 153	*n* = 30		*n* = 152	*n* = 30	
Soft tissue	0.0, 4.7 (0.0, 0.5)	0.0, 6.5 (0.0, 1.0)	0.104	6 (3.9)	2 (6.7)	0.388
	*n* = 155	*n* = 30		*n* = 155	*n* = 30	
Neurological	0.0, 8.0 (0.0, 0.5)	0.3, 6.1 (0.0, 1.3)	0.007	4 (2.6)	3 (10.0)	0.086
	*n* = 154	*n* = 30		*n* = 154	*n* = 30	
Function	0.0, 8.0 (0.0, 0.0)	0.0, 4.0 (0.0, 0.3)	0.006	2 (1.3)	4 (13.3)	0.007
	*n* = 155	*n* = 30		*n* = 155	*n* = 30	
Biobehavioral	0.4, 5.8 (0.0, 1.3)	0.8, 7.8 (0.1, 2.2)	0.032	8 (5.2)	4 (13.3)	0.109
	*n* = 154	*n* = 30		*n* = 154	*n* = 30	
Resource	0.0, 8.0 (0.0, 0.0)	0.0, 7.0 (0.0, 0.0)	0.852	5 (3.2)	1 (3.3)	0.981
	*n* = 76	*n* = 14		*n* = 76	*n* = 14	
Sexuality	0.0, 8.7 (0.0, 0.0)	0.0, 9.3 (0.0, 1.8)	0.306	8 (10.5)	2 (14.3)	0.651
	*n* = 155	*n* = 30		*n* = 155	*n* = 30	
Activity	0.0, 10.0 (0.0, 0.7)	0.0, 10.0 (0.0, 1.8)	0.031	16 (10.3)	5 (16.7)	0.345
	*Median, max (IQR)*	*Median, max (IQR)*				
	*n* = 157	*n* = 30				
No. of skin conditions in affected arm (max = 17)	0.0, 5 (0.0, 1.0)	1.0, 6 (0.0, 2.0)	0.009	n.a.	n.a.	n.a.

^a^ Mann–Whitney test.

^b^ Fisher exact test.

^*^ *p* < 0.05; ^**^p < 0.01.

na, not applicable.

**Table 6.** Summaries of Functional Assessment of Cancer Therapy Scores at Study Endpoint by Progression Group (*N* = 185)

FACT score	Scores		*p-*value*^a^*
	S-BCRL only (*n* = 155)	Progression to C-BCRL (*n* = 30)	
	Median (IQR)	Median (IQR)	
Physical (possible range: 0–28)^b^	26.0 (25.0, 27.0)	24.0 (20.5, 27.0)	0.002^**^
Social (possible range: 0–28)^b^	24.0 (21.0, 28.0)	25.0 (18.2, 28.0)	0.800
Emotional (possible range: 0–24)^b^	22.0 (19.0, 23.0)	20.5 (18.0, 23.0)	0.233
Functional (possible range: 0–28)^b^	23.0 (20.0, 27.0)	21.5 (13.7, 24.3)	0.027^*^
B-Subscale (possible range: 0–36)^b^	29.0 (25.0, 31.0)	26.5 (19.7, 30.3)	0.054
FACT-G Total (possible range: 0–108)^b^	94.5 (86.1, 101.8)	89.2 (72.6, 97.7)	0.021^*^
FACT-B Total (possible range: 0–144)^b^	121.2 (111.0, 131.8)	117.0 (95.0, 128.0)	0.027^*^
B + 4 (possible range: 0–16)^c^	0.0 (0.0, 1.0)	0.0 (0.0, 3.0)	0.140

^a^ Mann–Whitney test.

^b^ Higher score indicates better QOL.

^c^ Higher score indicates more B + 4 symptoms.

^*^ *p* < 0.05; ^**^p < 0.01.

At the study endpoint (either timing out of the study or progressing to C-BCRL), statistically significantly higher overall symptom scores were observed for the group of patients progressing to C-BCRL than for those who did not (*p* = 0.037), with 13.3% having overall scores ≥3 compared with only 1.3% in the group who did not progress (*p* = 0.007). Much of that overall symptom report difference can be accounted for specifically by a difference between the groups in LSIDS-A Function symptoms (*p* = 0.006). Similar to the overall scores, 13.3% of thoses who progressed had Function scores ≥3 compared with only 1.3% in the group who did not progress (*p* = 0.007; Table 5).

Specific symptom cluster score elevations for those who progressed compared with those who did not were also observed for the Neurological (*p* = 0.007), Biobehavioral (*p* = 0.032), and Activity (*p* = 0.031) symptom clusters. Of note, 16.7% (*n* = 31/155) of the entire sample reported scores ≥3 for the cluster of Activity symptoms, and within the subsample completing the Sexuality symptom scale, 11.1% (*n* = 10/90) (Table 5).

In addition, patients who progressed to C-BCRL for study endpoint had a significantly higher number of skin conditions than did the group that did not (*p* = 0.009; Table 5). One-third of patients who progressed (*n* = 10/30, 33.3%) had more than one skin condition, while only 18.5% (*n* = 29/157) had more than one in the group that did not progress.

Statistically significant differences between the groups in QoL were also observed at study endpoint (Table 6). Specifically, patients who progressed to C-BCRL had lower total Functional Assessment of Cancer Therapy-General and FACT-B scores, with specific differences in the Physical and Functional domains (all *p* < 0.05). No statistically significant difference was observed between the groups in FACT-B+4 scores (both median = 0.0, *p* = 0.140) (Table 6).

## Discussion

This is the first known study of newly diagnosed breast cancer patients undergoing intervention for S-BCRL initiated by either change in extracellular fluid or whole-arm volume to report symptoms, skin condition, and QoL findings at three specific time points: preintervention, immediate postintervention, and longer term end-of-study outcomes. Overall, at the time of S-BCRL detection, there was a low symptom burden; however, the LSIDS-A captured four clusters of problematic symptoms. These were in the activity, sexuality, function, and biobehavioral areas.

These findings demonstrate that S-BCRL is not an innocuous process. Immediately postcompression intervention, there were clinically meaningful reductions in patient-reported function (e.g., move arm side-to-side, raise arm above head) and biobehavioral symptoms (e.g., anger, appearance concerns); however, activity and sexuality scores were unchanged. It is possible that the activity (e.g., decrease social activity) and sexuality (e.g., lack of interest in sex) scores were driven by issues other than physiological S-BCRL that the intervention did not address.^21,22^ In patients with these symptoms, referrals for psychological support may be helpful.

Skin-related symptoms were also low, with examination identifying >1 condition in only 25% of the triggering sample. However, there was noted improvement in overall skin condition immediate postintervention. The maximum number reported was reduced from 9 out of a possible 20 symptoms to 6 out of a possible 20. This represents a meaningful clinical improvement. Although differences in skin conditions between groups were noted, during the time to study endpoint, few skin problems were observed. This suggests that compression intervention for S-BCRL may support skin integrity at a critical clinical time point.

QoL was generally high in this sample at the time of S-BCRL and intervention initiation. This is in keeping with the low symptom burden. In contrast with the LSIDS-A activity score that did not improve immediately postintervention, the FACT Physical score did improve, as did the lymphedema Breast +4 QoL items. This difference is likely explained by the LSIDS-A addressing more social activity constructs than that of physical activity itself.

Outcomes at the end-of-study time point were as expected. Those who progressed out of the study who were referred for management of C-BCRL had higher symptom scores than those who did not progress. These symptoms were primarily driven by the function cluster scores on the LSIDS-A. Function scores (e.g., move arm side-to-side, raise arm above head) improved immediately postintervention and remained improved except in those progressing, which supports the known symptom burden that accompanies C-BCRL.^3^ Biobehavioral scores, which did not improve immediately postintervention, were not elevated at study endpoint. This longer passage of time may have afforded patients the opportunity to cope/adjust to their overall cancer treatment and survival status.^23^ Those progressing to C-BCRL also had more skin conditions at end of study, and QoL scores were also lower than those who did not progress. Physical and functional domains in the FACT were the primary areas contributing to the lower scores.

Interestingly, the FACT-B+4 scores were similar between the two groups at study endpoint, indicating that patients who progressed were likely in early-stage C-BCRL and not experiencing severe symptoms. This finding may be due to the timely identification of C-BCRL afforded the patients undergoing prospective surveillance for early intervention in this study.

Higher level of change in L-Dex units at the time of S-BCRL detection correlated with higher overall symptom scores, as well as with higher soft tissue, neurologic, and sexuality symptom cluster scores. Similarly, only L-Dex also correlated with skin conditions at the same time point. These L-Dex findings are in keeping with prior research.^24^ No associations were found at that time with changes in total arm volume for either symptoms or skin conditions. L-Dex units also correlated with lower FACT physical scores, while arm volume correlated with higher FACT-B+4 scores, indicative of arm issues. Taken together, these findings primarily suggest that initiating prevention intervention using extracellular fluid change as a critical indicator for S-BCRL is warranted.

In addition, detection of extracellular fluid change in the presence of symptoms, as found in this study, supports S-BCRL as a clinically relevant construct. Further head-to-head comparisons of extracellular fluid change and arm volume change in symptom, skin, and QoL outcomes would be informative.

Clinical implications from this study are important. First, the instruments and methods used in this study were able to detect changes postintervention in this predominantly low symptom burden, high QoL sample. Thus, clinicians can use them with confidence in their practice. The findings confirm that, in clinical practice, ongoing patient self-report using valid tools, combined with identification of extracellular fluid change, provides actionable information to promote optimal timing of a compression intervention. It is recommended that patients receive ongoing, empowering education regarding the need to be aware and in-tuned to symptoms that they are experiencing throughout the prospective surveillance and early intervention model of care. Multidisciplinary breast cancer teams should be educated on these study results to inform patient education that optimizes outcomes for their patients.

### Strengths and limitations

This study includes an international sample of breast cancer survivors with results likely generalizable to newly diagnosed breast cancer patients in the United States, Australia, and other developed countries. Psychometrically sound self-report instruments were used, and longitudinal follow-up was almost 3 years postoperatively. The success of the intervention contributes to its primary limitation, a small number progressing to chronic lymphedema, limiting our ability to conduct multivariate analyses.

## Conclusions

Prospective surveillance, symptom assessment, and compression intervention promote low progression rates from S-BCRL to C-BCRL and as such reduce symptom burden.
